# Lead users’ ideas on core features to support physical activity in rheumatoid arthritis: a first step in the development of an internet service using participatory design

**DOI:** 10.1186/1472-6947-14-21

**Published:** 2014-03-22

**Authors:** Åsa Revenäs, Christina H Opava, Pernilla Åsenlöf

**Affiliations:** 1 Department of Neurobiology, Care Sciences and Society, Karolinska Institutet, Alfred Nobels Allé 23, 23100, Huddinge 141 83, Sweden; 2 Department of Neuroscience, Uppsala University, BMC, Box 593, Uppsala 751 24, Sweden

**Keywords:** Physical activity, Rheumatoid arthritis, Focus group, Internet service

## Abstract

**Background:**

Despite the growing evidence of the benefits of physical activity (PA) in individuals with rheumatoid arthritis (RA), the majority is not physically active enough. An innovative strategy is to engage lead users in the development of PA interventions provided over the internet. The aim was to explore lead users’ ideas and prioritization of core features in a future internet service targeting adoption and maintenance of healthy PA in people with RA.

**Methods:**

Six focus group interviews were performed with a purposively selected sample of 26 individuals with RA. Data were analyzed with qualitative content analysis and quantification of participants’ prioritization of most important content.

**Results:**

Six categories were identified as core features for a future internet service: up-to-date and evidence-based information and instructions, self-regulation tools, social interaction, personalized set-up, attractive design and content, and access to the internet service. The categories represented four themes, or core aspects, important to consider in the design of the future service: (1) content, (2) customized options, (3) user interface and (4) access and implementation.

**Conclusions:**

This is, to the best of our knowledge, the first study involving people with RA in the development of an internet service to support the adoption and maintenance of PA.

Participants helped identifying core features and aspects important to consider and further explore during the next phase of development. We hypothesize that involvement of lead users will make transfer from theory to service more adequate and user-friendly and therefore will be an effective mean to facilitate PA behavior change.

## Background

Despite growing evidence of the benefits of physical activity (PA) in individuals with rheumatoid arthritis (RA), the majority is not physically active enough [1-3]. The recommendations for individuals with RA are similar to those for the general population [4]. The difficulty in meeting recommendations might be due to the fact that PA is a complex behavior, determined by a number of bio-psycho-social factors [3,5-9]. As a consequence, various interventions to adopt and maintain PA behavior have been developed, e.g. supervised interventions in clinical settings and coaching of home-exercise programs [10-14]. These interventions report positive effects regarding general health perception, activity performance, aerobic capacity, muscle strength, disease activity and pain. However, long-term effects and maintenance of PA behavior have not been achieved [13,15].

Current evidence suggests that theoretically informed PA interventions delivered over the internet are effective in adult populations [16-20], in patients with chronic diseases and disabilities [21,22] and in children with juvenile idiopathic arthritis [23]. To our knowledge, there is only one previous study on internet-supported PA for people with RA, which also included face-to-face meetings and did not explicitly incorporate health behavior change strategies to guide the adoption and maintenance of PA [24]. Furthermore, its results indicated that integration of PA in participants’ everyday life was not achieved to any large extent [25].

An innovative strategy to increase the efficacy would be to move *from* a researcher-generated and health care-led design of PA interventions *to* one that include ‘lead users’ (i.e. potential users of the future service) in the process of designing the service. This user-centered approach, also known as participatory design [26], has frequently been applied to organizational development and design of health information systems [27,28]. More recently it has also been applied to design and re-design of health care services [29,30].

We intend to use participatory design to develop a theoretically informed and evidence-based internet service to support the adoption and maintenance of healthy PA behavior among people with RA. During the first step, we aimed to explore lead users’ ideas and prioritization of core features in a future internet service targeting adoption and maintenance of healthy PA.

## Methods

### Design and times of data collection

Six focus group interviews (FGIs) [31], were performed from October 2010 to March 2011. An inductive qualitative content analysis [32] was used to explore the participants’ ideas on core features. The qualitative analysis was complemented with a quantitative ranking of ideas to collect data on how the ideas were prioritized by participants.

### Participants and selection

Individuals diagnosed with RA [33] for no less than one year, 18 years or more of age and with adequate Swedish communication skills were eligible to participate. Potential participants were contacted by designated health care providers at three rheumatology clinics, one local hospital, one county hospital and one university hospital, in mid Sweden with both rural and urban catchment. An officer in charge of the membership directory at the Swedish Rheumatism Association (SRA) helped to select participants from their membership register. Participants were purposively selected to represent various ages, genders, years with diagnosed RA, PA behaviors, and internet experiences in order to capture variations in experiences and ideas.

After preliminary verbal consent, the first author (ÅR) contacted the potential participants by phone, providing verbal information about the study and collected verbal consent for participation. Written information was then sent out to those interested in participating and a total of 32 people accepted participation (n = 26 from hospitals and n = 6 from SRA). The Regional Ethical Review Board in Stockholm approved the study (D.nr. 2010/1101-31/5).

### Procedures and data collection

A preliminary semi-structured interview guide was developed and tested on four physiotherapy students. As a result, the guide was modified to comprise more thematic and open ended questions (Table 1).

**Table 1:** The semi-structured interview guide used during the focus group interviews

**Questions asked during the FGIs**	1. In your experiences, what enhances your physical activity?
	2. What ideas do you have concerning facilitating factors for physical activity in individuals with RA?
	3. What ideas do you have concerning important content on a website targeting physical activity in individuals with RA?
	4. How could these ideas appear on a website?
	5. In your opinion, which are the three most important experiences/ideas discussed today?
**Prompts and scenarios**^**1**^	*‘Can you tell me more about…’, ‘What did you think/feel then…’ ‘Pretend that a friend of yours has decided that she wants to be more physically active. She has never been a very active person. What should she do to start exercising? What advice would you give her?’*
	*‘Pretend that you just have gone through surgery and now you are back home. How do you get back to your old habits?’*

Note: FGI, Focus group interview; RA, Rheumatoid Arthritis.

^1^Examples of prompts and scenarios used to facilitate the discussion on participants’ actions, thoughts and feelings, and to cover situations on adoption and maintenance of physical activity.

The FGIs were conducted in undisturbed conference rooms at the rheumatology clinics or at the central office of the SRA. They lasted between 90 and 105 minutes and were audio-recorded. The FGIs were led by a moderator (first author; PhD-student with clinical expertise in physiotherapy and musculoskeletal conditions). One of two co-moderators (last author; associate professor with expertise in physiotherapy and behavioral medicine or a PhD-student with expertise in physiotherapy and public health) was also present to make prompts to develop the discussion, to take field notes, to provide feed-back to the moderator, and to assist in planning for the subsequent FGI based on experiences from the former. All interviews were transcribed verbatim by the first author in immediate connection to each FGI, i.e. during the next 1–3 days and before the performance of the next FGI. Initially we planned for four to five FGIs, but since new data were obtained during the fifth FGI it was decided to include one more. The sixth FGI did not result in any additional information, whereby the data collection was closed.

Each FGI started with the participants filling out questionnaires on background characteristics including readiness to engage in PA and internet habits. In the meantime, power-point scenarios illustrating PA in different contexts e.g. pictures of people mountain-biking, hiking, walking, gardening, playing soccer or doing water aerobics were shown to set the stage for the topic of discussion. Readiness to engage in PA was assessed with a self-report form [34] to indicate ‘maintenance phase’ (physically active for at least six months), ‘action phase’ (physically active less than six months), ‘preparation phase’ (plan to be physically active within one month), ‘contemplation phase’ (plan to be physically active within six months), or ‘precontemplation phase’ (do not plan to be physically active within six months). Internet experience was assessed with the question ‘Are you considering yourself a person comfortable using the internet? ‘-with answer options ‘-yes-’ or ‘-no-’. The participants were then informed about the aim of the study and their role in the design of a future internet service.

The set-up of the FGIs was inspired by the Nominal Group Technique [31]. Thus, before discussing the thematic areas, the participants had a few minutes for reflection on their own and to write down ideas on post-it notes. Thereafter, the participants shared what they had written to be discussed in the group. During the interviews, identified ideas were listed on a flipchart. To identify the participants’ opinions on the most important ideas to include in the future internet service, each participant ranked the three most interesting ideas listed, by assigning those three, two or one dots on the flipchart. To get a comprehensive summary of participants’ prioritizations, a list was compiled including the highest ranked ideas from each FGI, i.e. the four ideas receiving most dots. Ideas with similar content were clustered into one idea, resulting in a list with ten ideas of features in the future internet service. After completion of the sixth FGI, the list was mailed to all participants with a request for their votes on three core features that they would prioritize in the design of the future internet service.

### Data analysis

An inductive qualitative content analysis [32] was performed according to the following steps: *(1) Familiarization.* All transcripts were read to get an overview. *(2) Identification and extraction of codes.* Discussions on ideas of core features were identified, marked and cut out to a table. Meaning units with similar content received a unifying code. *(3) Comparison.* The codes were compared for identification of similarities and differences. *(4) Grouping.* Codes sharing similarities in content were sorted into subcategories. Next, subcategories reflecting similarities in content were sorted into manifest categories. *(5) Abstraction*. The categories were abstracted to themes reflecting the content areas important to consider during the next step of development. The relationship between meaning units, codes, subcategories, categories and themes is exemplified in Table 2.

**Table 2:** An example of the abstraction process from meaning units to codes, subcategories, categories and themes

**Meaning unit**	**Code**	**Subcategory**	**Category**	**Theme**
*FGI2*				
*(1)’… then you can sign up. Maybe there are others with RA interesting in the same thing who I don’t know that show up… We can meet*	- Advertise and schedule planned exercise	Contact with peers	Social interaction	Content
*(2)’It is like a meeting place on the web site*				
*(1) -Yes*				
*(2)- Then you can meet*				

*Note*: FGI (number) = Focus group interview, (number) = the participant.

To ensure credibility, researcher triangulation was used. The first and last authors read all transcripts, whereas the initial coding and sub categorizing were done by the first author. The coding was then presented to the last author who critically reviewed it with reference to her understanding of the transcripts. This resulted in analytic re-evaluation, revision of codes and sub categories, and formation of categories. The second author (Professor; expertise in physiotherapy and rheumatology) was introduced at the stage of categorization with the task to review the analytic track between codes, sub categories, and categories. This resulted in further revisions and refinements.

The quantitative data on participants’ prioritizations of core features were summarized and presented on a top 10 list.

## Results

Twenty-six out of 32 selected participants attended the FGIs, whereof 22 completed the prioritization task. The composition of the FGIs, including participants’ characteristics is shown in Table 3.

**Table 3:** Focus group composition and participant characteristics (n = 26)

**Focus group**	**1**	**2**	**3**	**4**	**5**	**6**	**Total**
	**SRA**^**1**^	**CH**^**2**^	**LH**^**3**^	**CH**	**UH**^**4**^	**LH**	**%**
	**n = 5**	**n = 6**	**n = 4**	**n = 4**	**n = 3**	**n = 4**	
**Gender**							
Male/Female	1/4	2/4	1/3	0/4	0/3	1/3	19/81
**Age**	46	65.5	57.5	37	54	69	60^5^
years	(31–63)	(63–71)	(54–62)	(31–50)	(41–64)	(68–71)	(31–71)
**Disease duration**	22	14.5	16	1.5	21	13.5	15^5^
years	(2–44)	(6–25)	(12–40)	(1–2)	(19–28)	(8–15)	(1–40)
**Marital Status**							
Married or Cohabitant/Single	2/3	6/0	2/2	1/3	3/0	1/3	58/42
**Working status**							
Full time	1	1	0	2	0	0	15
Part time	2	0	2	1	2	0	27
Retired	1	5	2	0	1	4	50
Other	1	0	0	1	0	0	8
**Education**							
University/High school/Public school	2/3/0	2/2/2	1/3/0	1/3/0	3/0/0	0/2/2	35/50/15
**Smoker**							
Yes/No/Previous	1/4/0	0/1/5	0/1/3	1/2/1	1/1/1	0/1/3	12/38/50
**Readiness to engage in PA**^**6**^							
Precontemplation	1	0	0	0	0	0	4
Contemplation	2	0	0	0	1	0	11.5
Preparation	0	0	0	0	0	0	0
Action	0	1	0	2	0	0	11.5
Maintenance	2	5	4	2	2	4	73
**Internet experiences**							
Yes/No	5/0	1/5	2/2	3/1	3/0	0/4	55/45

Note: ^1^*SRA*, Swedish Rheumatism Association; ^2^*CH*, County hospital; ^3^*LH*, Local hospital; ^4^*UH*, University hospital; ^5^Median (min-max); ^6^*PA*, Physical activity.

Data are shown as numbers with lowest and highest values within brackets.

### Ideas on core features in the future internet service: results from the FGIs

**Figure 1:**
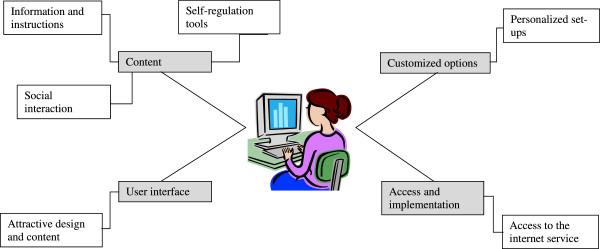
**The four themes reflecting the participants’ opinions on core aspects.** The picture included is a copy of Microsoft Word clip art with free access.

Participants’ ideas on core features concerned four aspects of the future internet service: (1) content, (2) customized options, (3) user interface and (4) access and implementation, see Figure 1. These aspects included the following categories: ‘up-to-date and evidence-based information and instructions’, ‘self-regulation tools to adopt and maintain PA behavior', ‘social interaction', ‘personalized set-up', ‘attractive design and content', and ‘access to the internet service’. The content of the categories are described below in narratives illustrating participants’ wishes, suggestions, considerations, and expectations on the new internet service as discussed during the FGIs (see also Additional file 1). In order to illustrate the entire range of variation ideas are presented without respect to how representative they were.

#### Content

##### Up-to-date and evidence-based information and instructions

This category refers to ideas on features for trustworthy information and instructions on PA adapted to the RA condition. Participants’ wanted inspiring movies, pictures and instructions on exercises and equipment. A keen discussion on whether movies and pictures should feature a typical person with RA or a brisk healthy person took place. Participants desired personal inspiring stories on how to succeed with PA as well as good examples of its impact on the disease and daily living. A question board for professional answers from physical therapists and physicians was suggested as well as inspiring news about PA in general, e.g. new training styles and trends. Participants emphasized the sense of being like anyone else with an interest in PA and valued PA news of general interest since it enhanced motivation. They regarded that up-to-date and evidence-based information on medication and its potential side-effects should be presented since effective medication was perceived a prerequisite for PA. Further, participants desired information about, and web-links to, fitness centers in the area providing adapted facilities and peer groups. They also wanted information about activities organized by the patient organization (SRA) and senior citizen organizations. The information should preferably be generated by users of the internet service. Particularly, information on fitness centers explicitly welcoming persons with disabilities was desired to avoid negative emotions of being unfit and disabled. Web-links to exercise programs on PA web-sites already existing were also suggested by participants.

##### Self-regulation tools

This category of ideas refers to tools assisting in the planning and performance of PA. Participants thought that planning and monitoring facilitated adoption and maintenance of PA. As a consequence they wanted possibilities to set personal goals and monitor performed PA in relation to those. A concrete idea was an electronic exercise diary with the possibility to keep track of performed exercise including notes on how it worked and was experienced. Another idea concerned display of diagrams on PA performed in relation to disease symptoms and well-being. The personal feed-back from such tools was expected to be inspiring and important for PA maintenance. Participants reasoned that these tools also could be used for planning of rehabilitation within the health care system. They also discussed how a personal web-based contract, including a description of PA mode, frequency and duration for a specific time period could help PA adoption. Feed-back on successful adherence was expected to result in personal satisfaction. One idea was to include performance tests to evaluate the effects of a PA program and to get feed-back on progression. Another was written instructions or a movie on range of motion tests.

Another discussion concerned the inclusion of personalized rewards. The act of rewarding was regarded as difficult and uncomfortable. Ideas about small tokens such as stars, points, or special visual effects were presented by participants. A concrete idea was to receive a piece of a jigsaw puzzle after having accomplished the exercise quota of the day to gradually create a nice picture as a result of success. Raffles with the chance to win books, theatre tickets or concert tickets were other proposals. It was vividly argued that the prizes should not be related to the disease, for instance something practical such as adapted scissors or bottle openers as this would only convey a sense of being ill and incapacitated. Participants emphasized the value of reminders, or cues for action, such as pop-up computer messages, short text messages or a smart-mobile application.

##### Social interaction

This category of ideas relates to features for connecting with other persons with RA. Participants proposed that chat groups and other possibilities for interactions should be included since they thought that the mere activity of chatting with peers about PA could be encouraging. They expected peers to come up with good exercises and information on where to find adapted facilities and groups. The participants also emphasized the value of discussions on how to handle possible side-effects of medication, which may hamper PA. They also hoped that social interaction would open up possibilities for finding new exercise peers. Exercise groups could be formed and planned activities scheduled and advertised on the web. An idea was the start of a dog agency, i.e. a notice board with information about dog owners in need of help with dog walks. The advocates thought that walking a dog would be much more fun than walking alone, and such an agency would be a creative PA facilitator.

#### Customized options

##### Personalized set-up

In this category, ideas on features for flexibility of the internet service to fit individual needs are compiled. The option to choose between levels of engagement was brought up by participants. Features for both “silent users” and for those mainly receiving information were suggested as were features for interaction possibilities. Concrete ideas for personalized set-ups were possibilities to create and save playlists for music. Participants wanted to be able to connect the personal exercise diary to Google calendar or Facebook. A smart phone application was another feature whished for.

#### User interface

##### Attractive design and content

This category of ideas concerns features for an enjoyable and inviting internet service, which participants’ perceived crucial for success. They underlined the importance of avoiding pointers and boring paragraphs on what should be done according to current PA recommendations. They wanted it to be fun and inspiring to log on to, for instance by the use of bright colors and photos from nice surroundings. One idea was to display surprises, e.g. frequently updated pictures and music on the front page.

#### Access and implementation

##### Access to the internet service

This category of ideas refers to features on how to get information about the internet service and how to be introduced to it. Participants were particularly concerned about how to reach the RA population. They thought that advertisements in local newspapers and in the SRA newsletter will be needed as will information brochures provided by primary health care and outpatient centers. A topic for discussion was the need of a personal introduction, provided by primary health care or hospital staff, to get started with the internet service. They also considered the possibility to introduce the internet service during postoperative rehabilitation while using the exercise programs available there. Access to the internet service already at the hospital or at a rehabilitation center may facilitate maintenance of PA behavior at home according to participants. They also brought up the possibility that the internet service would improve the implementation of evidence-based knowledge about PA among health care providers sharing exercise programs within the internet service. Another option discussed was the introduction of the internet service at the time of diagnosis, preferable provided by the physician in charge. They also wanted to be able to sign up for an introduction of the service via a link from the first page. A concern raised was that it may be difficult for some people with RA to adopt PA behavior without personal, face-to-face coaching. Still, participants expected these individuals to benefit from using the internet service for maintenance support.

### Prioritized core features to include in a future internet service

Table 4 illustrates the results of participants’ ranking of ideas. The top three core features were: (1) individual support strategies, (2) professional and peer advice about PA, and (3) information about exercise facilities and groups adapted to the needs of people with RA.

**Table 4:** Participants’ prioritization of ideas on features to include in the future internet service

	**FGI**^**1**^**1**	**FGI 2**	**FGI 3**	**FGI 4**	**FGI 5**	**FGI 6**
**The highest ranked ideas in each of the FGIs**	1. Rewards (7)^4^	1. Goal setting (13)	1. Incentives (9)	1. Find exercise peers (9)	1. Follow-up (5)	1. Exercise with peers (11)
	2. Training camps (7)	2. Time for PA^2^ (7)	2. Discussion foras (3)	2. Ask for advice about PA (3)	2. PA should be fun/incentive (3)	2. Specific exercise instructions (5)
	3. Adapted groups (4)	3. Free exercise (6)	3.Up to date information/research (3)	3. Find time for PA (3)	3. News on different PA (2)	3. Information about different PA (3)
	4. Learn how to reward one-self (4)	4. Exercise with peers (5)	4. Individual-ized exercise program (3)	4. Exchange experiences (2)	4. Individual-ized help (2)	4. Explanations about PA and RA^3^ (2)
**The top 10 list**	1. Individual support strategies (identify incentives, individualized exercise program, individualized help, specific exercise instructions)^6^*(11)*^5^					
	2. Advice about PA and RA from health care providers and peers (ask for advice about physical activity, exchange experiences) *(11)*					
	3. Information about exercise facilities and groups adapted to the needs of individuals with					
	RA, where to find exercise peers (training camps, adapted groups, find exercise peers, exercise with peers) *(9)*					
	4. Personal goal setting (set goals) *(8)*					
	5. Sharing experiences and ideas with similar others on PA related to RA (discussion foras) *(7)*					
	6. Individual follow-up of PA (follow-up) *(6)*					
	7. Up-to-date and evidence-based information and instructions on PA related to RA (information about different physical activities, explanation about physical activity and RA ) *(6)*					
	8. Ongoing research and news (up to date information and research, news on different PAs) *(6)*					
	9. Rewards (rewards, learn how to reward one-self) *(3)*					
	10. Individual support strategies to find time and routine for PA (routine, find time for physical activity) (0)					

*Note*: Free exercise (FGI 2) was not perceived possible to translate to ideas on features on the future internet service and therefore not included in the comprehensive prioritization and top 10 list.

Question asked for compilation of the top 10 list: ‘Rank the three most important core features to have access to on an internet service to support physical activity.’ One person voted on 2 ideas and 2 persons voted on 4 ideas.

^1^*FGI*, Focus group interview.

^2^*PA*, Physical activity.

^3^*RA*, Rheumatoid arthritis.

^4^*(n)* = The sum of dots from the participants’ ranking of the three most important ideas discussed during the focus group interviews.

^5^*(n) =* The sum of votes from the participants’ comprehensive prioritization of the three most important core features to include in the future internet service.

^6^(text) = the ideas from each FGI combined to the idea on the top 10 list.

## Discussion

This is, to the best of our knowledge, the first study involving individuals with RA in the development of an internet service to support the adoption and maintenance of PA. As a first step in the development process, the participants helped to identify core features important to consider during the next phases of development. The possibility for individual support strategies and to interact and get advice from professionals as well as peers was highly prioritized.

The participants stressed the importance of a trustworthy and inspiring *content*. One feature could be a question board with access to physicians and physical therapists providing credible advice, which has been associated with increased effect of internet services targeting PA in the general population [35]. Our findings also suggest that we should consider including personal experiential stories in the internet service. This was supported in a previous study reporting that such stories are helpful for the understanding of symptoms and course of disease [36]. Interestingly, normative information about others’ behaviors seems to enhance behavior change to a higher extent compared to provision of injunctive norms [36], which further confirms the importance of this aspect of content.

Self-regulation tools (e.g. self-monitoring) possible to relate to personal goals were emphasized by our participants. Self-monitoring combined with at least one additional behavior change technique increase the efficacy of PA interventions [37]. However, it is not yet clear whether tailored support is more effective compared to standardized support when provided via the internet, which needs to be further explored [19,38].

The possibility of peer interaction may contribute to the sense of mutual understanding and alliance. Shigaki and colleagues [39] evaluated an on-line interactive self-management program for individuals with RA and found that the included chat feature was little used, whereas the discussion board contributed to perceived support and bonding among participants. Another study reported that an on-line program for disease self-management had a positive impact on symptoms, health behaviors, self-efficacy and satisfaction with the health service as well as decreased utilization of health care [40]. This program was moderated by peers trained as expert patient program tutors, which might point to the importance of acknowledging peers as experts on their actual disease. We infer that this is a resource hitherto not utilized by health care to any significant extent, and therefore relevant to include in an internet service.

Our participants stressed the possibility for *customized options,* to attract the heterogeneous population of RA. Users of an internet service for disease self-management in individuals with RA have been reported to act in different ways depending on personal needs [41]. Some users may visit the internet service briefly to look for specific information whereas others log on to use available tools and search for social interaction. The need for a personal web profile for log on was discussed by our participants. However, too extensive procedures before getting access to the internet service can cause problem with low attendance, which has been reported elsewhere [20,42]. An open service has been suggested to enhance use [20,42]. Nevertheless, the possibility to choose level of engagement might be important in the new internet service to fit a variety of users.

Regarding the *user interface,* a positive appearance with no negative pointers was emphasized by our participants. The importance of a positive approach and uplifting stories has been described earlier by visitors of internet services for people with RA [36].

Our participants feared that the service would be difficult to disseminate and that there would be barriers for first-time users. To facilitate *access and implementation,* a personal face-to-face introduction was proposed. However, a recent review reported that internet services without face-to-face introductions were as effective as services with face-to-face contacts [19]. We infer that the availability of the internet service would be much higher without the need for a personal introduction. The participants’ opinion might have reflected concerns from those not comfortable using the internet, which raises important questions on whom the future internet service will be best suited for.

Incorporation of health behavior change theories is associated with more effective behavioral internet interventions [35]. However, evidence is limited on which principles to apply to reach the best effects [19,35]. A reflection on whether participants’ ideas corresponded to theoretical principles on adoption and maintenance of behaviors is therefore of value for the future development and evaluation of the service. According to socio-cognitive learning principles [43] behaviors are shaped through reciprocal interaction between the social context (e.g. behavior of peers) including the impact of role modeling, individual behaviors and prerequisites. Features proposed by the participants encompassing social interaction e.g. peer support, bonding, and inspiring stories of successful PA may all fit into this model. The participants also stressed the importance of up-to-date information and credible advice. This may increase motivation and intention to behavior change, but there is no firm support for the association between intention and actual behavior change [44]. They also provided many ideas on tools for goal setting and self-monitoring, which are both theoretically and empirically supported as means for self-regulation [37,45].

The unique feature and strength of our study is the involvement of lead users as the starting point for the design of a new internet service. Health care has for many years tried to involve patients into improvement of services, but it seems that patients are still not put first [46]. A strength of our study was the use of FGIs rather than individual interviews, which resulted in rich data. The discussions were vivid and participants inspired each other to come up with new ideas by listening to each others’ experiences. Another strength was the wide variation in participants’ education, age and disease duration which is assumed to be reflected in different opinions and ideas on how to adopt and maintain physical activity.

Challenges associated with user involvement include the decision on who should be selected and difficulties in recruiting users willing to participate in the often time consuming task, hence attrition of participants has been reported a great problem [27,29]. A limitation with our study was that the majority of participants already were physically active, and that the opinions of those below 31 years of age are missing, which possibly reduces the variation in ideas explored. In addition, 45% of the participants were not comfortable in using the internet and might thus not entirely represent the future target group. However, our intention in this first step of the development process was to collect a wide variation of ideas representing both those who were and were not comfortable internet users. Another issue is that those who declined participation due to the problem of attending the FGI may be the same persons needing flexible exercise routines, i.e. potential lead users of the internet service whose opinions were not explored. Individual interviews could have been a complement to the FGIs to capture the ideas of these individuals. Further, the transferability of our results are unknown at this stage of the project, hence one must not conclude that the ideas proposed and prioritized here are valid for the future target group. The transferability of the results from this first step of involvement of lead users must hence be tested in iterative cycles during the development of the internet service.

## Conclusions

Participants provided information on four aspects important to consider in a future internet service: (1) content, (2) customized options, (3) user interface and (4) access and implementation. High priority was given to content including individually tailored support for adoption of PA, advice from peers and health care professionals and information about adapted exercise facilities. Most of the ideas identified were possible to link to theoretical learning principles on how to adopt and maintain PA behavior changes. Nevertheless, we hypothesize that involvement of lead users will make transfer from theory to service more adequate and user-friendly and therefore will be an effective way to facilitate adoption and maintenance of PA.

We intend to make the lead users not only a reference group but *co-designers* of the future internet service. The next step in the development process will be to set up a series of workshops where lead users, health care providers, researchers, and web-designers collaborate to produce a requirement specification for the internet service. This will include studies on the collaboration and decision-making process, refinement of content and interface and specification of system requirements.

## Competing interests

The authors declare that they have no competing interests.

## Authors’ contributions

ÅR and PÅ were involved in the design, data collection, analysis and manuscript writing. CO participated in the design, analysis and manuscript writing. All authors read and approved the final manuscript.

## Pre-publication history

The pre-publication history for this paper can be accessed here:

http://www.biomedcentral.com/1472-6947/14/21/prepub

## Supplementary Material

Additional file 1 Overview of content analysis on codes, categories and categories. Click here for file
