# Supplementary material for: Lead users’ ideas on core features to support physical activity in rheumatoid arthritis: a first step in the development of an internet service using participatory design
Source: BMC Med Inform Decis Mak. 2014 Mar 22;14:21. doi: 10.1186/1472-6947-14-21 (PMC3998038; doi:10.1186/1472-6947-14-21)
Supplement: Additional file 1 — Overview of content analysis on codes, categories and categories. [file 1472-6947-14-21-S1.pdf]

Additional file 1. Overview of content analysis on codes, subcategories and categories.

Code	Subcategory	Category
- Adapted PA	Movies and pictures	INFORMATION AND INSTRUCTIONS
- Exercises and equipment		
- A typical person with RA or a brisk healthy person		
- Personal, inspiring stories		
- Ask a physical therapist or a physician	Advice	
- Training styles and trends	News on exercises in general	
- Medication and potential side-effects	Medication	
- Web-links to organizations and fitness-centers	Survey available resources	
- Information from peers		
- Web-links to PA web-sites		

Note: PA=Physical activity, RA=Rheumatoid arthritis, FGI=Focus group Interview, (number)=the participant

Additional file 1 cont.

Code	Subcategory	Category
- Monitor performance and progression	Planning and monitoring	SELF-REGULATION TOOLS
- Electronic exercise diary		
- Diagrams on performed PA		
- A web-based contract		
- Written instructions or a movie	Performance tests	
- Personalized	Rewards	
- Learn how		
- Collect small tokens		
- Special visual or audio effects		
- Collect pieces of a jigsaw puzzle		
- A raffle		
- On computer or mobile phone	Cues for action	
- Chat groups	Share experiences and ideas	SOCIAL INTERACTION
- Tips on good exercises		
- Find exercise facilities and groups		
- Handle side-effects of medication		
- Find exercise peers	Contact with peers	
- Form exercise groups		
- Advertise and schedule planned exercises		
- A notice board to borrow a dog	Dog agency	

Note: PA=Physical activity, RA=Rheumatoid arthritis, FGI=Focus group Interview, (number)=the participant

Additional file 1 cont.

Code	Subcategory	Category
- Information or interaction	Flexible in use	PERSONALIZED SET-UPS
- Personal- web-profile		
- Music play-lists		
- Links to Google calendar or Facebook	Links to applications	
- A smart-phone application		
- No pointers or boring articles	Enjoyable and inviting	ATTRACTIVE DESIGN AND CONTENT
- Bright colors and pictures		
- Display surprises		
- Advertisements	Information	ACCESS TO THE INERNET SERVICE
- Information brochures		
- Primary health care or hospital staff	Personal introduction	
- Postoperative rehabilitation		
- At time of diagnosis		
- Sign up on the first page of the web-site		

Note: PA=Physical activity, RA=Rheumatoid arthritis, FGI=Focus group Interview, (number)=the participant
